# Consumer-Grade Electroencephalogram and Functional Near-Infrared Spectroscopy Neurofeedback Technologies for Mental Health and Wellbeing

**DOI:** 10.3390/s23208482

**Published:** 2023-10-15

**Authors:** Kira Flanagan, Manob Jyoti Saikia

**Affiliations:** 1 Electrical Engineering, University of North Florida, Jacksonville, FL 32224, USA; 2 Biomedical Sensors and Systems Laboratory, University of North Florida, Jacksonville, FL 32224, USA

**Keywords:** neurofeedback, EEG, fNIRS, wellbeing, mental health

## Abstract

Neurofeedback, utilizing an electroencephalogram (EEG) and/or a functional near-infrared spectroscopy (fNIRS) device, is a real-time measurement of brain activity directed toward controlling and optimizing brain function. This treatment has often been attributed to improvements in disorders such as ADHD, anxiety, depression, and epilepsy, among others. While there is evidence suggesting the efficacy of neurofeedback devices, the research is still inconclusive. The applicability of the measurements and parameters of consumer neurofeedback wearable devices has improved, but the literature on measurement techniques lacks rigorously controlled trials. This paper presents a survey and literary review of consumer neurofeedback devices and the direction toward clinical applications and diagnoses. Relevant devices are highlighted and compared for treatment parameters, structural composition, available software, and clinical appeal. Finally, a conclusion on future applications of these systems is discussed through the comparison of their advantages and drawbacks.

## 1. Introduction

Neurofeedback training began as a study of consciousness through the elicitation of alpha brain wave activities (associated with relaxation). Using a reward-based system, a study noted that subjects were able to increase their production of alpha amplitudes over time by correctly determining what brain wave stage they were in [1]. Neurofeedback is a form of self-regulation based on physiological variables that are modified by the individual that were previously thought to be involuntary. Significant interest in these noninvasive applications led to studies of the efficacy of neurofeedback training for epilepsy, attention deficit disorder, hyperactive disorder, and later, anxiety and mental wellness [2].

Advancements in the field of neuroscience have led to the proliferation of brain-mapping devices, including functional magnetic resonance imaging (fMRI), electroencephalography (EEG), functional near-infrared spectroscopy (fNIRS), and magnetoencephalography (MEG), in order to expand and deepen our understanding of the neural mechanisms underlying various psychological and neurological conditions. Neuroplasticity research has also provided new insights into the mechanisms that underlay neurofeedback training, such as showing the connectivity of the regions of the amygdala and hippocampus during training and how to improve neural synchrony [3].

Consumer neurofeedback devices are increasingly being used for mental health monitoring and treatment. These devices are based on EEG and fNIRS technology. These devices are portable, affordable, and can be used outside of traditional clinical settings, making them more accessible to a wider range of clients. However, there are challenges to consider.

The quality and reliability of data are important factors when indirectly measuring neuronal activity. Signals produced by firing neurons and collecting signals on the scalp are often affected by movement, noise, and signal interferences, and as a result, the data collection can be marred by noise and be difficult to interpret. Additionally, the subtlety of brain activity changes necessitates improvements in the technology employed by consumer devices. Consumer devices are not governed by any set protocols. Standardization is another critical consideration, as the absence of established protocols hinders result comparisons across multiple studies and poses barriers to clinical utilization.

State-of-the-art technology is beginning to address some of these challenges. For example, machine learning algorithms can be used to improve the quality of deciphering EEG and fNIRS signals by filtering out noise and identifying patterns that are relevant to mental health and wellbeing monitoring [4,5]. Newer EEG devices are also incorporating additional sensors, such as eye-tracking or heart rate monitors, to provide more comprehensive data and an inertial measurement unit (IMU) to measure and correct motion artifacts. Additionally, efforts are underway to establish standardized protocols for data collection and analyses, which will make it easier to compare results across studies and establish the best practices for clinical use.

### Purpose

**Figure 1:**
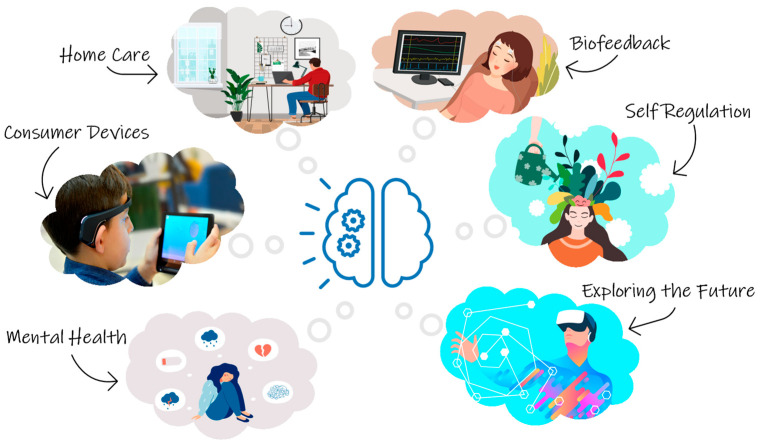
Consumer neurofeedback devices and their opportunities for at-home care, biofeedback training, mental wellbeing regulation, and future virtual reality (VR).

In this review paper, we discuss relevant wearable neurofeedback devices currently on the market, particularly in the consumer space. Our focus is specifically on consumer-grade EEG and fNIRS devices that are designed for use by the public. These devices aim to bring neurofeedback training and brain activity monitoring outside of traditional clinical settings and make them accessible to a wider range of individuals. Figure 1 presents the applications and opportunities of consumer neurofeedback devices, such as for at-home care, biofeedback training, mental wellbeing regulation, and virtual reality (VR). By utilizing these consumer-grade devices, individuals can engage in self-regulation and optimize their brain function in the comfort of their own homes or other non-clinical environments. We compared their design and recognition approach to data processing. In addition to the review of consumer neurofeedback devices, this paper also includes an analysis of relevant studies conducted in the field. These studies contribute to the understanding of the efficacy and potential applications of neurofeedback devices in the context of mental wellbeing. By examining the findings and methodologies of these studies, we gain insights into the effectiveness of consumer-grade EEG and fNIRS devices in treating various mental health disorders and improving overall wellbeing.

Furthermore, this paper explores the current advancements and drawbacks in the field of mental wellbeing associated with consumer neurofeedback devices. It discusses the progress made in terms of device design, treatment parameters, and available software. The advancements in technology, such as incorporating additional sensors for comprehensive data collection and employing machine learning algorithms for data processing, are also highlighted. On the other hand, the paper acknowledges the existing challenges and limitations of consumer-grade neurofeedback devices. Issues related to data quality, signal interference, and standardization are discussed. The current landscape of these devices has seen significant progress, with established evidence supporting neurofeedback, especially in clinical samples. However, a look into the standardized protocols to further enhance the clinical utility could be addressed in order to see a continuing widespread adoption of these techniques. By critically examining these drawbacks, the paper aims to provide a balanced perspective on the current state of consumer neurofeedback devices and their potential for mental wellbeing applications.

## 2. Wearable Neurofeedback Technologies

**Figure 2:**
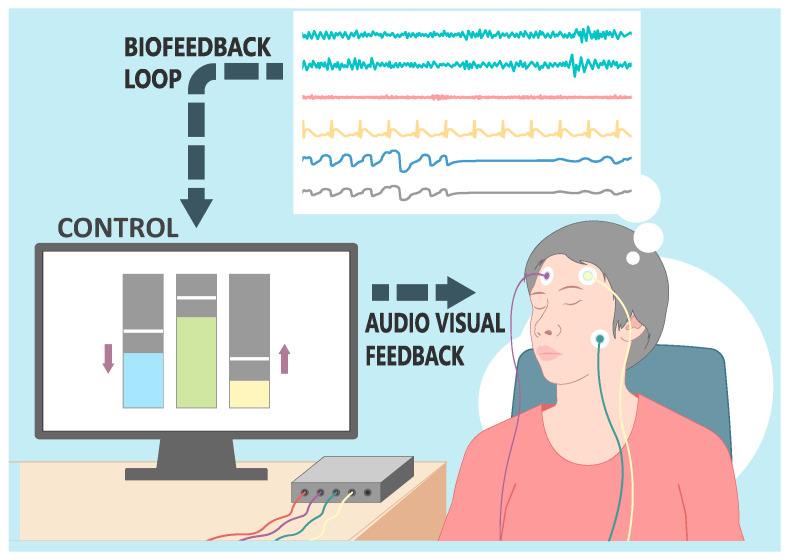
Illustration of a real-time biofeedback system. The image captures a female participant outfitted with an EEG head cap. Visible on the display are the dynamic brainwave patterns. These patterns are processed in real-time, with key oscillatory metrics extracted and fed into a control system. The control subsequently modulates audio–visual feedback being presented to the participant, establishing an interactive biofeedback loop.

In the following sections, we will provide an overview of commercially available EEG and fNIRS devices designed for consumer use. We have extensively researched commercially available devices, and here we compare their relevant specifications, including the number of channels, sampling rate, electrode type, and connectivity options for EEG devices, as well as the number of channels, wavelengths used, sampling rate, and sensor array configurations for fNIRS devices. Additionally, we will discuss the data collection, signal processing techniques, and sensor arrays employed by these devices, highlighting their key components and features. Figure 2 will serve as a visual summary of the components in a neurofeedback system, illustrating the interplay between data collection devices, signal processing algorithms, and the stimulus.

### 2.1. EEG Consumer-Grade Devices

**Figure 3:**
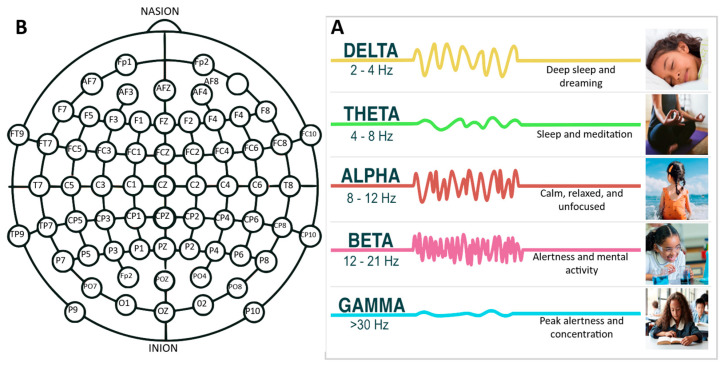
(**A**) A detailed schematic representation of electrode placements across the scalp, highlighting standardized locations. This section delineates common configuration points that are essential for ensuring consistent and comparable data across research and clinical studies. (**B**) An informative chart elucidating various EEG frequency bands—delta, theta, alpha, beta, and gamma—and their associated cognitive and physiological activities. This component serves to emphasize the distinct brain activities and states associated with each frequency range.

Electroencephalography (EEG) is a measurement of the brain’s voltage potentials from the postsynaptic activity of neurons and of the cortex via electrode arrays. In numerous studies, there are key parameters and categories that experts frequently emphasize, including aspects of sampling frequencies capabilities and signal processing functionalities that are tailored for specific research goals. Sampling frequency, a category that refers to how often the EEG records data within a second, is an important construct. A higher sampling frequency enables the capture of increasing high-frequency oscillations of the brain’s activity. EEG systems often incorporate specialized signal processing functionalities. These functions filter, refine, or amplify raw data to isolate specific brainwave patterns and phenomena, such as the K complex, which are distinctive waveforms often observed during sleep stages. Detecting and understanding such patterns is paramount in studies pertaining to sleep disorders or cognitive processes during rest. These signals can be used to generate an understanding of the correlation of the cognitive processes of attention [6,7], memory [8], language [9], and emotion [10,11,12,13,14]. The delineation between raw EEG data and the derivative insights obtained from Quantitative EEG (QEEG) is an important construct. While EEG affords a raw electrocortical snapshot, QEEG delves deeper, furnishing an analytical framework that underpins individualized neurofeedback therapeutic strategies. A key advantage of EEG is its high temporal resolution, where continuously monitored brain activity can be captured on the order of milliseconds [15]. A system with a sampling rate of 500 Hz records EEG data 500 times every second. This enables it to detect even fleeting changes in brain activity, essential for understanding rapid neural responses. For certain research and clinical scenarios, where precise timing and rapid responses are crucial, such as the detection of seizure onset in epilepsy, higher sampling frequencies become indispensable. Furthermore, when analyzing these signals, there are prominent characteristics that are isolated and examined for the determination of a cognitive or neurological state. For example, the K complex is a hallmark of non-REM (rapid eye movement) sleep and can indicate sleep quality and disturbances. EEG can focus on the oscillations of electrical activity that occur at different frequency bands, such as alpha waves, which occur at a frequency of around 8–12 Hz [16]. As shown in Figure 3B, these frequency bands are associated with particular brain activities and mental states. In addition to research, EEG has a variety of clinical applications [17], such as diagnoses and tracking of various neurological [18] and psychiatric conditions [19], which are briefly mentioned in this review paper.

EEG devices offer several advantages over other brain imaging techniques, such as functional magnetic resonance imaging (fMRI) and positron emission tomography (PET), which involve ionizing radiation and contrast agents. Consumer-grade EEG devices are portable, user-friendly, and cost-effective compared to fMRI and PET technologies, making them suitable for various settings, including schools, home care, and field work. Table 1 illustrates the affordability and accessibility of EEG devices compared to their counterparts. Overall, EEG serves as a versatile and valuable brain-imaging tool, providing safe insights into brain functionality.

**Figure 4:**
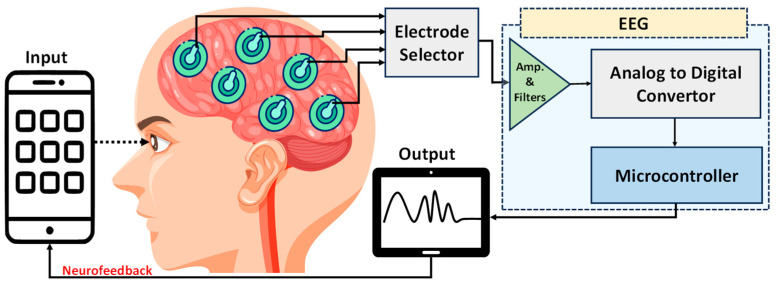
Conceptual diagram of an EEG neurofeedback system. Electrodes placed on the scalp capture neural activity, which is amplified and filtered. These analog signals are converted to digital by an analog-to-digital converter, processed by a microcontroller, and then relayed back to a smartphone for real-time feedback. The design illustrates the closed-loop nature of contemporary EEG feedback systems.

Consumer electroencephalographic devices commonly consist of a sensor array, an amplifier, and an interface for data acquisition and analysis, as shown in Figure 4. A biomedical sensor array is characterized by a group of sensors or electrodes laid in a geometric pattern, used for collecting electrical, electromagnetic, electrochemical, acoustic, optical signals, etc., from the human body. In the case of EEG, the accurate placement of the electrode over the scalp or cortex is important in order to obtain comprehensive and accurate measurements of brain electrical activity. The sensor arrays used in these devices can vary in terms of the number and placement of electrodes, as well as the types of additional sensors included. Fixed, flexible, multimodal, and wireless array types are commonly used. These arrays may be incorporated into lightweight, flexible caps made of materials such as silicone or neoprene or integrated into molded headset configurations.

In EEG, channels correspond to the specific electrical connections established between electrodes positioned on the scalp and other anatomical sites, as presented in Figure 3A. Each channel represents the electrical activity between two electrodes and can be used to detect and measure different aspects of brain activity [20]. The number and arrangement of channels in EEG devices may vary depending on the specific device and intended application. Some EEG systems may comprise a limited number of channels, while others can incorporate dozens or even hundreds of channels. Generally, a higher number of channels provides more comprehensive and accurate measurements of brain activity. Notably, consumer-grade EEG devices commonly employ reference electrodes, which serve as electrophysiological constant reference potentials for measuring electrical activity within brain tissue [21]. These reference electrodes enable a comparison of the voltage potentials across different electrode locations with respect to a common reference point, facilitating the acquisition of precise and reliable data.

EEG systems typically consist of sensor and electrode arrays that are applied to the scalp using a conductive gel or solution. These arrays can encompass a range of electrodes, with some systems accommodating up to 256 electrodes positioned around the head, as depicted in Figure 3A. However, commercial EEG products generally employ fewer electrodes, commonly adopting configurations such as the 10–20 system, with 19 or 21 electrodes, or the 10–10 system, with 64 or more electrodes spaced at shorter intervals [22,23,24,25]. Each channel of these systems corresponds to a specific region on the scalp, with electrodes placed accordingly and labeled to indicate their respective locations. It is important to note that not all channels on an EEG device are dedicated to measuring brain electrical activity; some are used for capturing additional physiological signals such as heart rate, electrooculogram (EOG), or muscle activity. For example, select channels can be tailored to record the electrooculogram (EOG), which measures eye movement and can be crucial for studies involving rapid eye movement during sleep or visual tracking tasks. Similarly, channels detecting heart rate can provide insights into the interplay between cognitive processes and cardiac responses, offering a holistic understanding of certain experimental conditions or clinical scenarios. Moreover, it should be acknowledged that the scalp regions shown in Figure 3A can exhibit considerable variation depending on the specific experimental or clinical parameters being considered.

Although consumer EEG devices hold promise for elucidating brain activity and mental states, it is crucial to recognize that these devices lack medical-grade certification and should not be employed for clinical diagnosis purposes unless subjected to rigorous validation and regulatory approvals. Moreover, careful interpretation of the collected data is warranted due to potential limitations in signal quality. Sampling rate problems can arise, impacting the accuracy of the captured signals, as consumer devices often operate at lower sampling rates compared to medical-grade EEG systems. This lower sampling rate may result in an incomplete representation of the underlying neural activity and may hinder the detection of rapid changes or high-frequency components. Furthermore, the noise levels inherent in consumer EEG devices can be significant, stemming from various sources such as suboptimal electrode placement, inadequate electrode–scalp contact, inherent device limitations, motion artifacts induced by user movement, and environmental noise [26]. These noise factors introduce distortions into the acquired signals, thereby diminishing the fidelity of the recorded data and potentially complicating the interpretation of neural activity patterns [27].

### 2.2. fNIRS Consumer-Grade Devices

**Figure 5:**
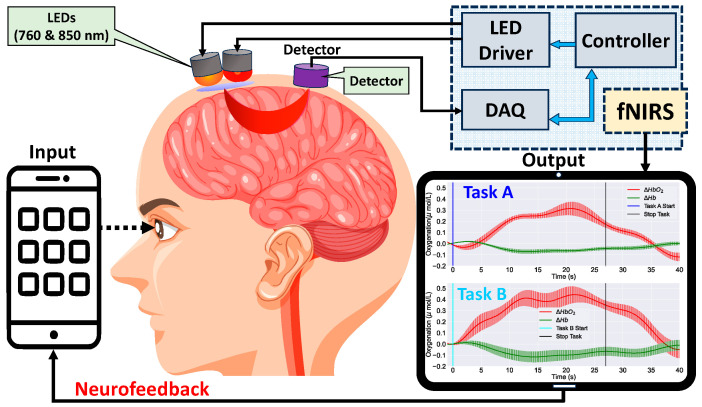
Conceptual diagram of an fNIRS neurofeedback system. Optodes positioned on the scalp emit and detect near-infrared light to measure cerebral blood flow changes. The acquired data are processed and digitized, then sent to a microcontroller, which in turn relays information back to a smartphone for real-time feedback. This representation underscores the closed-loop design of modern fNIRS feedback systems.

Functional near-infrared spectroscopy (fNIRS) is a non-invasive imaging application that utilizes optics and NIR light (e.g., 760 and 850 nm wavelengths) to penetrate the scalp and skull to reach brain tissue. When the emitted light reaches the brain, it is absorbed by the oxygenated and deoxygenated hemoglobin in the cortical area of the brain. The absorption ability depends on the oxygenated level of hemoglobin, which is a proxy for blood flow to the activated brain regions [28,29,30]. The detected light on the scalp using light detectors is then used to calculate the changes in blood oxygenation levels to determine neural activity [31,32]. Figure 5 presents the conceptual diagram of fNIRS. Similar to EEG, fNIRS also has several advantages over fMRI, PET, and MEG. It is less sensitive to motion artifacts, has a higher temporal resolution compared to fMRI, and is more portable and affordable [33,34,35]. fNIRS is increasingly being used in various applications, including cognitive neuroscience [36,37,38], sleep studies [39], clinical research, brain–computer interfaces, and neurofeedback [40,41].

While fNIRS devices can be more common than other modalities, they are still costly and not as pervasive as EEG devices. However, fNIRS offers several advantages over electroencephalography (EEG) in neuroimaging. Firstly, fNIRS provides greater spatial resolution, allowing us to pinpoint brain activity with superior precision. Since fNIRS can detect changes in the oxygenated and deoxygenated hemoglobin concentrations within specific brain regions, it offers a more localized view of neural activity. Its lower susceptibility to movement artifacts also makes fNIRS suitable for studies requiring participants to be in naturalistic environments. A significant advantage of fNIRS is the absence of conductive gels and electrodes on the scalp, which minimizes setup times and improves participant comfort. The cost of fNIRS devices can range from a few thousand to tens of thousands of dollars depending on the type of device and its capabilities. This cost variation is influenced by several parameters such as the number of channels, types of detectors, and processing software capabilities that accompany the device. Institutions and research organizations must weigh these factors carefully against their budget constraints when considering an fNIRS device for purchase. For example, devices equipped with a larger array of channels to cover a broader area of the brain tend to be at a higher end of the price range. These high-channel configurations are often required for advanced neurological studies that need to capture a comprehensive set of data points from different brain regions. These types of devices all require proper training as well in order to interpret the data, often necessitating specialized expertise or even certified courses. This training not only encompasses how to operate the device but also delves into data analysis, ensuring that findings are accurate and not subject to common pitfalls or misinterpretations. Despite their growing popularity, fNIRS devices remain uncommon in comparison to the fMRI systems available in clinical settings and the EEG devices available for both clinical and commercial purposes. It is worth noting that fMRI and EEG technologies have a longer history, providing them with a more extensive user base and a wider range of applications. The longevity of these technologies has also resulted in a more extensive knowledge base, extensive troubleshooting insights, and a broader range of training resources. The technology still remains in the early stages of development, and this is paired with a smaller market [42]. Many fNIRS devices featured in studies are based on the author’s own design and not on a prefabricated device, which indicates both a level of specialization in the research community and a certain adaptability of the technology. This bespoke approach also signifies the current phase of the technology, where many researchers are still experimenting and optimizing the device’s design for their specific needs.

However, even with the scarcity of commercial fNIRS devices, there is a growing number of companies that are manufacturing and selling these devices. These companies are spearheading the drive to bring fNIRS to both the research community and potentially the broader consumer market. Much like the wide range of EEG devices on the market, fNIRS devices can come with varying capabilities, different channels, multiple detectors, and differing spatial resolutions. This emerging marketplace indicates that fNIRS technology is maturing, suggesting that as competition grows and technology improves, there may be a drive towards more standardized, user-friendly, and potentially more affordable options in the future. A few companies to consider are Artinis, NIRx Medical Technologies, Mendi, and Obelab.

In fNIRS devices, the light source and detectors are arranged in specific source/detector patterns called channels, as seen in Table 2. Each channel consists of a multi-wavelength light source and one or more detectors, and the signals from each detector are combined to estimate the changes in blood flow in the underlying brain tissue [33]. This multi-wavelength approach is pivotal because different wavelengths can penetrate tissues to various depths, allowing researchers to gather data from different layers of the brain. By analyzing the absorption rates of these wavelengths, researchers can make informed conclusions about brain activity and blood oxygenation. The number and placement of channels can vary depending on the specific fNIRS device and application, but typically, there are anywhere from a few to several dozen channels. High-channel devices, designed for advanced research scenarios, offer a more comprehensive coverage of the cerebral cortex, enabling a nuanced understanding of both localized and networked brain activities. Additionally, short separation channels are a relatively newer format of densely packed sensors, meant to characterize more artifact activities in order to improve the resolution of the signals.

fNIRS devices have their own strengths and limitations to consider. These devices have a higher spatial resolution and the ability to localize activity to specific regions of the brain, positioning them as a powerful intermediary tool that blends some of the advantages of both fMRI and EEG devices. This means that while they may not offer the full spatial resolution of fMRI devices, they can give more detailed spatial information than EEG devices. fNIRS has proven to pick up activity in deeper regions [43]. This depth of penetration, while not as deep as fMRI, offers insights into the subcortical regions, providing valuable data that are not easily obtained with surface-level EEG electrodes. While the cost of the majority of fNIRS devices is lower, EEG equipment is commonly accepted and widely available due to its straightforward and well-established data analysis interfaces [44]. The EEG’s legacy in neuroimaging research and its simplistic yet effective functionality make it a staple in many labs. However, the increasing sophistication of fNIRS data-processing tools is starting to bridge this gap, making fNIRS a more accessible and appealing choice for a wider range of researchers. There is limited availability on the market, with the available consumer devices being relatively new and not widely available to consumers. However, they have several limitations, including their limited spatial resolution and signal contamination issues. As technology continues to develop, fNIRS may become a valuable tool for home and clinical usage.

### 2.3. Data Acquisation

Data acquisition refers to a process of collecting raw data from a specific source in a controlled and systematic procedure. In neuroscience research, data acquisition typically features specialized devices, such as EEG or fNIRS devices, that measure electrical and optical signals generated by the brain. There are several steps involved that include the placement of sensors to clean scalp and calibration to optimize the signal-to-noise ratio.

EEG devices are equipped with an array of electrodes that measure the differential amplification of thousands of postsynaptic potentials produced in the brain in microvolts (μV). The voltage fluctuations measured are diminutive and require a sensitive data acquisition (DAQ) system comprising an amplifier and analog-to-digital converter (ADC). The amplifier is designed to increase the signal amplitude by a factor of several thousand through a high-gain and low-noise amplifier circuit for detection and usable data. An important characteristic of EEG amplifiers is the input impedance, which is a measure of the opposition to current in a static and dynamic form to determine signal behavior. Scalp–electrode interfaces have a relatively high impedance, which requires a high input impedance at the amplifier to avoid the distortion and attenuation of signals. Noise performance is another important characteristic due to the nature of the microvolt signals being received to limit the introduction of additional noise [13,45,46,47]. This can be limited through the design of the circuit, noise isolation, and the quality of the components used. Another aspect of the DAQ system is the characteristics of the ADC, such as the bit resolution and sampling rate of the ADC, which also influence the quality of the EEG signal and the maximum frequency of the EEG signal. Therefore, through strategic attention to the design and optimization of the DAQ system, researchers can effectively address challenges related to signal quality in EEG recordings. By implementing noise isolation measures, utilizing high-quality components, and selecting suitable ADC characteristics, the recorded EEG data can exhibit improved accuracy, reliability, and the capability to capture signals across a wide frequency range.

### 2.4. Signal Pre-Processing

This section will review the series of techniques and methods that are applied to raw data to improve their quality, remove artifacts, and prepare them for further analysis. Both EEG and fNIRS are susceptible to various types of noise and artifacts, including muscle activity, eye movements, environmental noise, electrode artifacts, and signal penetration. As an example, muscle activity can introduce high-frequency noise in EEG data, while motion artifacts are a common issue in fNIRS, often necessitating specialized techniques for each modality.

Preprocessing techniques are used to minimize the impact of noise to the enhance signals of interest in EEG and fNIRS devices. The overarching aim here is to obtain a signal that accurately represents the neural or hemodynamic activity under study, isolating it from non-neural factors that could compromise the integrity of the data. Common preprocessing techniques for these devices include filtering, artifact removal, re-referencing, epoching, baseline correction, and types of Fourier transforms. The choice among these techniques often hinges on the specific goal of the analysis; studies focusing on event-related potentials in EEG might prioritize epoching and baseline correction, whereas fNIRS studies looking at sustained cognitive activity might employ sophisticated filtering techniques. Filtering involves the attenuation of specific frequency ranges from the signal. This is particularly important when investigating phenomena that are known to occur at specific frequencies, such as alpha waves in EEG or the slower oscillations related to hemodynamic responses in fNIRS. High-pass filters can be used to remove low-frequency noises such as Mayer waves and slow drift, while a low-pass filter can be used to remove high-frequency noises such as the removal of sudden physiological movements from fNIRS signals [48]. For EEG, high-pass filters are often critical when the aim is to study fast neural oscillations, such as gamma waves. For fNIRS, removing high-frequency noise can be crucial when the focus is on slower hemodynamic changes. Another common noise, 50/60 Hz line noise, is often removed using a notch filter in the case of EEG. This is universally important for any EEG study because electrical noise from the environment can profoundly skew the data. This is less of a concern for fNIRS data, which typically focus on slower oscillations that are far removed from the frequencies of electrical noise. There are additional techniques involved in cleaning this raw data, such as a Principal Component Analysis (PCA), which is designed to eliminate artifacts from EEG data [49]. PCA can be particularly effective when the goal is to separate multiple sources of neural activity or to distinguish neural signals from noise. It operates by transforming the original variables into a new set of variables, the principal components, which are orthogonal (uncorrelated) and reflect the maximum variance. Epoching refers to the segmentation of the continuous EEG signal into shorter segments known as epochs to isolate and analyze specific events. This is often essential in studies that are event-related, such as those investigating responses to stimuli, where the neural activity of interest occurs within a brief time window following the stimulus. A baseline correction is the removal of the average activity during a baseline session from each time interval in the signal in order to remove issues of drift. This is useful in both EEG and fNIRS when you want to normalize the signals to a reference point, making it easier to compare activity across different time points or conditions. A wavelet transform (WT) is a time-frequency mathematical analysis that breaks a signal into its constituent wavelets at different frequencies. Wavelet transforms are beneficial when the frequency content of a signal changes over time, as they can provide information on both the frequency and the time at which specific frequencies are present. This is highly valuable for analyzing non-stationary signals such as human EEG or fNIRS data. A small, oscillating signal is used to analyze a much larger signal via discrete or continuous functions [50,51]. While this format is an improvement to the Fourier transform, it has a sensitivity to noise, making it difficult to extract meaningful information without redundancy. The wavelet transform is often used in EEG for identifying changes in oscillatory activity over time, such as during sleep stages, or in fNIRS when studying rapid changes in oxygenation levels during cognitive tasks.

## 3. Neurofeedback Pattern Recognition

This section will review patterns in data via discrete recognition by trained models. Neurofeedback devices use this approach as a basis for understanding that specific patterns of activity within the brain are associated with specific mental states, such as relation, focus, and anxiety. The inherent goal of discrete recognition is to train an individual to produce specific patterns of brain activity that are otherwise deemed involuntary.

### 3.1. Signal Processing

Power spectral density is a widely used technique that utilizes autoregressive modeling to analyze the power distribution across different frequency bands [52]. It offers enhanced frequency resolution and allows for targeted improvement in specific frequency ranges [53,54]. Commonly considered frequency bands include delta, theta, alpha, beta, and gamma [55]. Each of these frequency bands has significance in the realm of brain activity. The delta band is associated with deep sleep and is dominant in infants. The theta band is often linked to creativity, relaxation, and drowsiness, while the alpha band is related to relaxed alertness. The beta band is observed during active, analytical thought, and the gamma band is connected with complex cognitive tasks, information processing, and problem solving. Different brain research or neurofeedback applications might emphasize one or more of these bands. In terms of equipment, a higher sampling rate, typically at least twice the highest frequency of interest (based on the Nyquist theorem), is needed to capture gamma frequencies adequately. For instance, to study gamma frequencies, a minimum sampling rate of 200 Hz would be recommended.

The number of sensors required depends on the spatial resolution needed for the study. While studying global patterns of brain activity, such as sleep stages (which heavily feature delta waves), fewer sensors might suffice. In contrast, when studying cognitive tasks that activate specific brain regions (e.g., gamma activity in the prefrontal cortex during problem solving), more sensors and precise placement are necessary to achieve granular data. As for signal attenuation, this largely depends on the depth and region of the brain being monitored. Surface electrodes might be sufficient for capturing alpha rhythms from the visual cortex, but deeper structures would require signal amplification and more sensitive equipment. Consumer devices, which are often designed for more general uses, might not offer the granularity of specialized research equipment. Companies such as Emotiv or Muse, for instance, provide EEG headsets with multiple channels suitable for basic frequency analysis, but more detailed studies might require advanced lab-based setups.

Coherence, on the other hand, is a measure of the synchronization between signals originating from two distinct brain regions. It is employed to estimate the reliability of peaks in a signal and effectively suppress spikes caused by noise [56]. The assessment of linear dependency between two signals at a given frequency is crucial in coherence analyses, and correlation coefficients are commonly used to quantify the degree of similarity.

Event-related potentials (ERPs) are small voltage fluctuations that occur in response to physiological or cognitive events. These potentials arise from the coordinated summation of postsynaptic potentials during information processing [20,57,58,59,60]. ERPs are valuable for studying brain responses related to specific stimuli or tasks and provide insights into cognitive and perceptual processes.

By employing appropriate signal processing techniques, researchers can extract meaningful features from brain signals, facilitating a deeper understanding of brain functioning and cognitive processes. The choice of preprocessing methods depends on the specific goals and requirements of the research study or application.

### 3.2. ML/AI Classification

Mathematical or machine learning (ML) algorithms are used to classify brain signals into categories [61,62,63,64]. The process is used to accurately distinguish data into different states, tasks, or conditions based on patterns and specific characteristics observed by EEG or fNIRS signals. When using this method, relationships between target features and target categories are determined and then tested or trained on novel data [65,66].

There are several types of classification methods that are used with EEG and fNIRS signals, including linear discriminant analysis, support vector machines, artificial neural networks, and deep learning methods. These methods can be used to classify features in EEG sub-bands, delta (0.5–4 Hz), theta (4–8 Hz), alpha (8–13 Hz), beta (13–30 Hz), and gamma (>30 Hz) [67], as featured in Figure 3B. In an fNIRS analysis, the signals are not categorized by frequency bands, as they are based on the changes in oxygenated and deoxygenated hemoglobin concentrations. As a result, fNIRS signals are sorted into task-related activation patterns based on spatiotemporal characteristics [68].

The selection of the appropriate classification algorithm often hinges on the nature of the data, their dimensionality, and the kind of features extracted. For EEG signals, which are temporal and encompass multiple frequency bands, the spectral power within these bands or the phase coherence between different sensors can be considered as features. For fNIRS, hemodynamic responses or task-evoked concentration changes in oxygenated and deoxygenated hemoglobin serve as primary features. It is worth noting that the spatial resolution of EEG is generally lower than fNIRS. Thus, sensors such as high-density EEG arrays, offering increased spatial granularity, might be preferable when discerning minute spatial patterns. In contrast, fNIRS, benefiting from its relatively higher spatial resolution, might employ optodes placed over the regions of interest, particularly when analyzing task-specific activations.

Linear discriminant analysis (LDA) is a supervised learning algorithm designed to find a linear combination of features that maximize the separation between classes onto a low-dimensional space. This is accomplished using discriminant functions that maximize the ratio of between-class variance and within-class variance. This is an easy-to-implement system that can operate on high-dimensional data using kernel methods. Support vector machines (SVMs) are another variance of supervised learning computation used for the separation and filing of data. They differs from LDA in that SVMs find a hyperplane of high-dimensional data that parses out the limits of different groupings in the input data [69]. SVMs tend to be less sensitive to outliers due to the increased focus of this maximal separation of data. This property makes SVMs favorable for EEG signals where non-linearities often exist, especially with high-dimensional feature sets obtained from many sensors. Furthermore, SVMs’ resilience to outliers ensures robust performance even when the EEG data contain artifacts or external interferences. Artificial neural networks (ANNs) are another popular method of machine learning, inspired by the formation and positions of the network of neurons in a human brain. The method consists of multiple layers, where the input layer receives the data, and the output layer produces the classification. The hidden layers perform nonlinear transformations that build on the growing history of the network. ANNs, due to their adaptability and depth, can model complex and high-dimensional data structures, making them ideal for capturing intricate patterns in EEG or fNIRS signals. They especially shine when the relation between input features and output classes is non-linear or when the data structure has hierarchical patterns. However, the success of ANNs requires sufficient training data, and for brain–computer interface applications or real-time neurofeedback systems, ensuring timely and effective training of the network is pivotal.

## 4. Software Applications

There are multitudes of available software and applications for consumer EEG and fNIRS devices that allow a user or professional to record, analyze, and visualize brain activity. Popular software for EEG and fNIRS devices include OpenBCI, BrainWave, Muse Direct, NIRS Toolbox, and COBI Studio.

OpenBCI provides open-source software for recording and analyzing EEG data. The company hosts headsets, sensors, boards, and electrodes for neurofeedback and brain-computer interfacing. OpenBCI has the benefit of flexibility with clear results using a scientifically validated research platform. Muse Direct is software that leans towards consumer-oriented meditation [70,71,72]. The software is user-friendly, but the systems usually lack the number of channels and electrodes available. Given its consumer-friendly approach, Muse Direct is generally not equipped for nuanced clinical diagnostics or complex neurofeedback protocols necessitating medical supervision. BrainWave is similar to OpenBCI but features advanced visualization settings that can detect isolated signals in up to 4096 electrodes. Despite the advanced visualization offered by BrainWave, its applicability for rigorous scientific research or clinical intervention should be validated through peer-reviewed studies. Most consumer devices on the market use their own built-in applications and software that feature games, meditation, electrical signal output, and performance, as seen in Table 3. While these platforms grant a considerable degree of autonomy and customization, it is essential to underscore the nuanced differences between self-guided neurofeedback and the supervised, calibrated approach espoused by professionals. The former, though accessible and user-friendly, may occasionally fall short in terms of accuracy and therapeutic efficacy.

There are a variety of open software programs available online for various consumer EEG devices, but there are also many phone- or web-based applications for not only monitoring brain activity but also a consumer’s mental state and cognitive performance.

Muse features a smartphone app called “Muse: Meditation & Sleep” that offers real-time feedback on brain activity with guided meditation sessions. The app relies on sound to indicate the different states in the brain, such as calm and active. The goal is to train the user to recognize when they enter a meditative state, much like in the original Nowlis et al. study from 1970. The app features meditation sessions that are designed to improve stress, focus, and sleep. There are goals and challenges available to keep user engagement and motivation high. Sleep tracking is another benefit that makes personalized recommendations for sleep improvement.

Consumer EEG and fNIRS devices are primarily designed to strengthen mental processes through improved attention [73], memory [74], and decision making. However, the key limitation here is that ‘improvement’ is often gauged through built-in metrics that may not align with clinically accepted indicators. Depending on the particular goals of those utilizing these electronic devices, their benefits and objectives could shift. The most significant claimed benefit to utilizing these programs is improved attention, as they teach consumers how to maintain attention and focus for extended periods of time. Improved attention is an expansive phrase that encompasses a variety of cognitive processes that are involved in maintaining attention through periods of focus. There is statistical significance that EEG training can improve attention in the short term for individuals with attention deficit hyperactivity disorder [75,76,77,78].

A study aimed at evaluating the impact of mobile neurofeedback applications on children’s cognitive performance had a group size of 37 and a testing duration of four weeks. The application focused on self-regulation tasks to reduce anxiety through a game interface. The results of the study found that the children had a statistically significant improvement in attention and regulation as opposed to the control group [79]. While these findings are promising, it is important to underscore the need for long-term follow-up studies under controlled, clinical settings to determine the sustainability of such improvements.

The positive effect on attention performance that utilizes neurofeedback training is likely due to the neuroplasticity characteristics of the brain, according to several studies [80,81,82]. High plasticity indicates that information processing and capacity are trainable [83]. By relying on this basis of neuroplasticity, EEG and fNIRS devices can provide instructive feedback conditioning through computerized cognitive training, video games, and mobile training apps [84,85,86]. An increase in self-awareness through operant conditioning in neurofeedback training can also train recognition in an individual when their focus shifts from a task [87]. These training exercises in neural connectivity that underlay the brain’s adaptivity can potentially allow for positive long-term changes in maladaptive behavior.

## 5. Mental Health and Wellbeing

There is a growing industry in EEG and fNIRS devices in mental health and clinical practice [88]. These devices have traditionally been used in research settings to study brain activity and aid in diagnosing neurological conditions [89]. However, with the introduction of affordable, consumer-grade devices, interest in the potential towards mental health applications has risen. Yet, it is crucial to distinguish between the capabilities of consumer devices and the expertly calibrated tools in a professional setting. Consumer EEG and fNIRS devices can be used as a supplement to professional medical treatment for anxiety and depression and other mental health issues, but not as a tool for diagnoses. Instead, professionals can utilize the information gathered to assist them make a diagnosis and arrange effective therapy.

Treatments for various health issues can also be checked at home by specialists using these neurofeedback devices. They can be used to study changes in brain activity over time, offering vital information into the success of various therapies for mental health issues [4,90]. However, unsupervised usage can lead to misinterpretation of data or missed critical signs, highlighting the indispensable role of therapists, specialists, or psychologists in this domain. Monitoring brain activity after psychotherapy or drug treatment for depression and anxiety is one example. Biofeedback therapy, a procedure involving providing real-time feedback to individuals regarding physiological responses to stressors, can be employed with EEG and fNIRS devices. The therapy can allow for a patient to learn to regulate their responses to stressors using visual or auditory feedback via a computer program [91]. During sessions, individuals receive feedback on brainwave activity and are encouraged to make adjustments to their behavior or thought patterns in response. Hence, therapists, specialists, and psychologists play a vital role in this process by designing and administering individualized neurofeedback protocols tailored to the individualized needs and goals of their clients. They are skilled at interpreting neural and behavioral data and helping clients understand the link between brain activity and emotions. Furthermore, therapists provide emotional support and guidance throughout the training, helping clients manage their psychological and emotional reactions to neurofeedback. Psychologists also contribute by assessing the effectiveness of the neurofeedback protocol and adjusting protocols as necessary to ensure optimal outcomes. Their expertise in psychological assessment and therapy techniques enhances neurofeedback training, improving mental health and wellbeing.

One study looked into the use of EEG neurofeedback for patients suffering from serious depression. Patients who received 30 sessions of neurofeedback training over 10 weeks demonstrated substantial changes in mood and reduced depressive symptoms when compared to the control group [92]. In a different study, generalized anxiety disorder patients’ anxiety symptoms were evaluated in conjunction with the impact of EEG training. The study found that patients who received neurofeedback training showed significant reductions in anxiety symptoms compared to the control group due to the self-regulation of brain activity based on a classical training strategy that lasts long after therapy [93].

A study primarily focused on the alpha frequency band (8–12 Hz) found that neurofeedback training improved mental agility in healthy adults, with the greatest improvements observed in response inhibition and cognitive flexibility [16]. Another study discovered that neurofeedback training in the theta frequency band (4–8 Hz) increased mental agility in people with attention deficit hyperactivity disorder (ADHD), with the largest benefits in reaction inhibition and working memory [94,95,96,97,98,99,100,101,102,103].

The mechanics underlying how neurofeedback training relieves depression and anxiety symptoms are not fully understood. One possible explanation is that neurofeedback training can alter the brain circuits involved in emotion regulation, resulting in improved emotional processing and moods [104]. However, while these studies showcase promising outcomes, they underscore a salient point: the pivotal role of supervised neurofeedback training from a licensed specialist. While the findings of these trials can be encouraging due to their statistical significance, more research is still needed in order to determine the utility and impact of consumer neurofeedback devices in treating mental wellbeing issues.

## 6. Discussion and Conclusions

Consumer devices for EEG and fNIRS are tools marketed for promoting relaxation, reducing stress, and improving an individual’s attention and focus. These devices are not intended for clinical or research use and are not approved as a method of treatment for mental health conditions. There is evidence that biofeedback therapy can be helpful in treating depression, anxiety, and attention disorders, but the scientific evidence supporting the use of these consumer neurofeedback devices is still limited.

### 6.1. Challenges

Consumer-grade EEG and fNIRS devices hold considerable promise for transforming mental health screening and treatment. Having observed the rise of consumer-grade devices, we recognize the growing appetite for such technology. This trend, while exciting, does necessitate a grounded perspective. However, they face notable challenges in their current state, primarily concerning the accuracy, reliability, interpretability, wearability, and data quality standards of these devices.

In comparison to their research and clinical counterparts, consumer-grade devices exhibit lower levels of accuracy and reliability. From a personal standpoint, the allure of convenience these devices offer contrasts sharply with the demands for precision in mental health assessment. The precision and accuracy of the collected raw data can be influenced by various factors, including the quality of electrodes and other components. Additionally, the interpretation of the collected data relies on human expertise, which can be intricate and specialized. The wearability aspect of these devices can also pose limitations, as headbands equipped with numerous electrodes may be uncomfortable for users. This echoes the earlier sentiment that the user experience, while vital, should not overshadow the core function: accurate data collection.

A significant challenge lies in the quality and reliability standards of the data captured by consumer devices. Both EEG and fNIRS are indirect measures of brain activity, rendering their signals susceptible to disturbances such as motion artifacts, environmental noise, and signal interference. Consequently, the obtained data may contain noise artifacts and pose challenges in interpreting subtle shifts in the neuronal activity related to an individual’s psychological state. Such discrepancies highlight the need for continuous advancements and rigor in device development.

The absence of standardized guidelines for data collection and interpretation in the context of mental health applications is a pressing concern. Personal reflections upon this underscore the importance of a collective push for standardization, bridging the divide between consumer- and clinical-grade devices. The lack of consensus regarding protocols hampers the comparison across studies and the establishment of clear clinical guidelines.

Furthermore, ensuring information confidentiality and security is a crucial aspect [105,106]. Consumer-grade EEG and fNIRS devices typically collect sensitive personal data, including brain activity information, which, if mishandled, could be exploited for malicious purposes. In an era where data privacy is paramount, it is ever more crucial that these devices uphold the highest standards of data protection. Building and maintaining user trust in these devices and their associated applications necessitates robust data security measures and privacy safeguards. Notably, the growing accumulation of user data in large databases for machine learning applications raises both opportunities and concerns regarding the analysis of brain activity and mental health.

Addressing these challenges requires concerted efforts from researchers, developers, and regulatory bodies to enhance the accuracy, interpretability, wearability, and data quality of consumer-grade EEG and fNIRS devices. With collaborative effort and adherence to rigorous standards, we might bridge the gap between the potential and actual utility of these devices. Establishing standardized protocols, improving data collection techniques, and implementing robust security measures are vital steps toward realizing the full potential of these devices in advancing mental health research and practice.

A number of the aforementioned concerns are now being addressed by cutting-edge technology. The continued use of machine learning methods, for example, can be used to improve the quality of EEG and fNIRS data by filtering out noise and discovering patterns related to mental health. Newer EEG devices are also incorporating additional sensors, such as eye tracking [5] or heart rate monitors, to provide more comprehensive data. There are efforts currently underway to standardize data collection and analysis techniques, which will make it easier to compare results across research studies and create the best practices for clinical application.

To summarize, while consumer-grade EEG and fNIRS sensors show great promise for mental health applications, there are still considerable difficulties to overcome. Modern technology is addressing some of these issues, but considerable effort needs to be made to guarantee that these devices are dependable, effective, and secure for use in mental health monitoring and treatment. Drawing from the present landscape, it becomes evident that while we are on the precipice of a technological revolution in mental health, there is much groundwork yet to be carried out.

### 6.2. Future Direction

The future of consumer neurofeedback devices features developments and advancements in several key areas. As technology continues an upward trend of improvement, the likelihood of consumer EEG and fNIRS devices becoming more accurate and reliable with regards to measuring brain activity increases. This possibly can lead to far more precise and targeted therapies for biofeedback.

Accessibility is a current selling point for consumer neurofeedback devices, with most features on the market being relatively affordable and portable. Advancements in technology and the democratization of mental health care can make them more accessible to a wider demographic of consumers. Devices such as Muse, OpenBCI, and NeuroSky are already available in the market and are relatively affordable compared to traditional neurofeedback equipment. The widespread use of smartphones and other mobile devices could potentially allow for a large-scale dissemination of neurofeedback training to people who would not have access to traditional neurofeedback equipment. Furthermore, advancements in machine learning and artificial intelligence could lead to the development of individually tailored and adaptive neurofeedback training programs.

The addition of other devices or sensors are a current researched topic. Devices such as fitness trackers or smart watches can build a bigger profile for an individual’s mental and physical wellbeing. Virtual reality (VR) can also provide potential applications for the treatment of phobias or stress disorders. VR enables the creation of immersive and interactive environments that can increase user engagement during neurofeedback training while also providing more realistic and ecologically valid training scenarios [107]. Several studies have looked into the use of virtual reality in conjunction with consumer neurofeedback equipment. A particular investigation explored adopting a VR-enhanced neurofeedback system to improve attention among adolescents experiencing attention deficit/hyperactivity disorder (ADHD). The study demonstrated that children who underwent VR-enhanced neurofeedback training improved their concentration considerably in comparison to children who received non-VR neurofeedback instruction [108]. Another study investigated the use of a VR-enhanced neurofeedback system for training relaxation skills in patients with anxiety disorders. When compared to a control group that received non-VR neurofeedback training, patients who received VR-enhanced neurofeedback training perceived a greater decline in anxiety symptoms [109].

Virtual reality (VR) has been a promising avenue in the realm of consumer neurofeedback devices for training emotional regulation skills. Using consumer neurofeedback devices has several advantages. VR can provide a more engaging and realistic training environment, which can enhance the motivation and interest of users during training. Additionally, VR can provide a more ecologically valid training scenario, which can help users generalize their learned skills to real-life situations.

## Figures and Tables

**Table 1:** A comparison of popular EEG devices for consumers: electrode configuration, sensor type, headset design, and applicable audience.

Manufacturer/Model	Channels	Reference Channels	Configuration Type	Additional Sensors	Audience
BrainRobotics BrainLink Lite	4	Yes	Headband	Accelerometer, gyroscope	Consumer industry, mental wellbeing
EMOTIV EPOC FLEX	>32	Yes	Cap	Gyroscope, accelerometer, magnetometer, PPG, temperature	Research and development, university, consumer industry
EMOTIV INSIGHT	5	Yes	Headband	Gyroscope, accelerometer, magnetometer, temperature	Consumer industry
EMOTIV EPOC+	14	Yes	Headband	Gyroscope, accelerometer, magnetometer, temperature	Research and development, university, consumer industry
INTERAXON Muse 2	4	Yes	Headband	PPG, accelerometer, gyroscope, magnetometer, temperature	Meditation coaches and consumer industry
MACROTELLECT Brainlink Pro	5	No	Headband	Accelerometer, gyroscope, magnetometer, temperature	Advanced education health and mental wellbeing
MYNDPLAY Myndband	1	Yes	Headband	Accelerometer, gyroscope, magnetometer, temperature	Advertising, virtual reality, research and development
NEEURO Senzeband 2	4	Yes	Headband	Accelerometer, gyroscope, temperature	Performance training
NeuroSky MindWave Mobile 2	1	Yes	Headband	Accelerometer, gyroscope, temperature	Beginner EEG developers
FocusCalm	5	Yes	Headband	Accelerometer, gyroscope, temperature	Performance training
EMOTIV EPOC X	14	Yes	Headband	Accelerometer, gyroscope, magnetometer, temperature	Research and development, university, consumer industry
Interaxon Muse S	5	Yes	Headband	Accelerometer, gyroscope, PPG, breath, temperature	Beginner EEG developers
OpenBCI EEG Kit	8	Yes	Headband	--	Research and development, instructors, students

**Table 2:** A comparison of popular fNIRS devices on the market: channel types, sensor types, headset design, and applicable audience.

Manufacturer/Model	Channels	Short Separation Channels	Configuration Type	Additional Sensors	Audience
NIRx NIRSport2	8–80	Yes	Cap	Pulse oximetry, heart-rate variability, oxygen saturation, respiration, temperature, galvanic skin response	Research and development, clinical use
Artinis Brite MKIII	<27	Yes	Cap	Gyroscope, accelerometer	Research and development, university, consumer industry
Artinis PortaLite MKII	7	Yes	Headband	Gyroscope, accelerometer	Research and development, university, consumer industry
Mendi	2	No	Headband	--	Meditation coaches and consumer industry
Obelab NIRSIT	48	No	Headband	Gyroscope, accelerometer	Research and development, university, consumer industry
Obelab NIRSIT Lite	15–19	No	Headband	Accelerometer, gyroscope, compass	Research and development, university, consumer industry

**Table 3:** An outline of the available software for the selected EEG and fNIRS devices.

Manufacturer/Model	Device Type	Application
EMOTIV EPOC FLEX	EEG	EmotivPro
EMOTIV INSIGHT	EEG	“Emotiv BCI, MyEmotiv, BrainViz, Mental Commands”
EMOTIV EPOC+	EEG	EmotivBCI, MyEmotiv
INTERAXON Muse 2	EEG	Muse Direct
MACROTELLECT Brainlink Pro	EEG	Macrotellect software, not compatible with Neurosky
“MYNDPLAY Myndband”	EEG	Misc. third party software
“NEEURO Senzeband 2”	EEG	Mindviewer
“NeuroSky MindWave Mobile 2”	EEG	Visualizer, third party software
FocusCalm	EEG	FocusCalm
EMOTIV EPOC X	EEG	EmotivBCI, SDK Cortex
Interaxon Muse S	EEG	Muse Direct
BrainRobotics BrainLink Lite	EEG	Macrotellect software
Mendi	fNIRS	Mendi.io
NIRx	fNIRS	Turbo-Satori, Aurora fNIRS
Artinis Medical System	fNIRS	OxySoft
Obelab	fNIRS	Obelab Connect, third party software

## Data Availability

No new data were created or analyzed in this study. Data sharing is not applicable to this article.
